# Psychological distress and associated factors of the primary caregivers of offspring with eating disorder during the coronavirus disease 2019 pandemic

**DOI:** 10.1186/s40337-021-00405-9

**Published:** 2021-04-29

**Authors:** Lei Zhang, Meng Ting Wu, Lei Guo, Zhuo Ying Zhu, Su Fang Peng, Wei Li, Han Chen, Juan Fan, Jue Chen

**Affiliations:** Shanghai Mental Health Center, Shanghai Jiao Tong University School of Medicine, 600 WanPing South Road, Shanghai, 200030 China

**Keywords:** COVID-19, Eating disorder, Primary caregivers, Depression, Anxiety, Perceived stress, Social support

## Abstract

**Background:**

The Coronavirus Disease 2019 (COVID-19) is a global pandemic and posed serious challenges in many countries. A number of studies before the COVID-19 pandemic have shown that the primary caregivers of the ED patients are subjected to great burden, psychological pressure, and serious emotional problems. This study aimed to investigate the psychological distress level of the primary caregivers of ED offspring during the COVID-19 pandemic.

**Methods:**

From March 6 to April 20, 2020, 378 questionnaires for primary caregivers of ED offspring and 1163 questionnaires for primary caregivers of healthy offspring were collected through an online crowdsourcing platform in mainland China. Valid questionnaires that met the criteria included 343 (90.74%) primary caregivers of ED offspring and 1085 (93.29%) primary caregivers of healthy offspring. Using Propensity Score Matching (PSM), 315 (83.33%) primary caregivers of ED offspring and 315 matched primary caregivers of healthy offspring were included in the statistical analysis. Depression, anxiety, perceived stress and social support were measured by Patient Health Questionnaire-9, Generalized Anxiety Disorder-7, Perceived Stress Scale-10 and Social Support Rating Scale, respectively.

**Results:**

The rates of depression and anxiety of the primary caregivers of ED offspring were 20.6 and 16.5%, which were significantly higher than those of primary caregivers of healthy offspring (4.1 and 2.2%), all *P* < 0.001. Regression analysis found that perceived stress, social support, previous or present mental illness, family conflicts during the COVID-19 pandemic had a significant impact on the severity of depression (*P* < 0.001, *P* = 0.002, *P* = 0.041, *P* = 0.014); Perceived stress, social support, family conflicts during the COVID-19 pandemic and years of education had a significant impact on the severity of anxiety (*P* < 0.001, *P* = 0.002, *P* = 0.002, *P* = 0.003).

**Conclusions:**

During the COVID-19 pandemic, primary caregivers of ED offspring experienced more psychological distress than that of primary caregivers of healthy offspring. ED caregivers with high perceived stress may have higher levels of depression and anxiety. ED caregivers with high social support, no mental illness and no family conflicts may have lower levels of depression. ED caregivers with high social support, no family conflicts, and high years of education may have lower levels of anxiety.

## Plain English summary

The Coronavirus Disease 2019 (COVID-19) is a global pandemic and posed serious challenges in many countries. This study aimed to investigate the psychological distress level of the primary caregivers of eating disorder (ED) offspring during the COVID-19 pandemic, and to explore potential influencing factors. From March 6 to April 20, 2020, 378 questionnaires for primary caregivers of ED offspring and 1163 questionnaires for primary caregivers of healthy offspring were collected through an online crowdsourcing platform in mainland China. All primary caregivers were evaluated for depression, anxiety, perceived stress and social support. 315 primary caregivers of ED offspring and 315 matched primary caregivers of healthy offspring were included in the statistical analysis. We compared the primary caregivers of ED offspring with the primary caregivers of healthy offspring for depression, anxiety, perceived stress and social support. We found that primary caregivers of ED offspring experienced more psychological distress than that of primary caregivers of healthy offspring. ED caregivers with high perceived stress may have higher levels of depression and anxiety. ED caregivers with high social support, no mental illness and no family conflicts may have lower levels of depression. ED caregivers with high social support, no family conflicts, and high years of education may have lower levels of anxiety.

## Background

A novel coronavirus emerged in Wuhan, China, at the end of 2019, and rapidly spread to other parts of China. By March 2020, the Coronavirus Disease 2019 (COVID-19) has become a global pandemic and posed serious economic, political, social, and health challenges in many countries [1]. In order to slow down the rate of transmission of COVID-19, many countries have adopted quarantine policies, such as closing schools, factories and other public places. Governments encourage people to maintain social distance by staying at home. This has slowed down the virus spreading to some extent, but it also has an impact on people’s mental health. Stressors including but not limited to longer quarantine duration, infection fears, frustration, boredom, inadequate food, and inadequate information, have increased people’s negative feelings such as depression and anxiety [2].

Eating Disorder (ED) refers to a group of mental disorders characterized by abnormal eating behaviors and psychological dysfunction, accompanied by significant weight changes and/or physical dysfunction, mainly including anorexia nervosa (AN), Bulimia Nervosa (BN) and Binge Eating Disorder (BED) [3]. Affected by the quarantine policy, most patients with ED do not have access to clinical assessment and treatment services, and ED symptoms may be aggravated by food shortages, food insecurity and fear of infection [4, 5]. In particular, there is a lack of eating disorder treatment centers in China, and some of which were unable to provide offline clinical assistance after the COVID-19 outbreak and did not have a systematic and comprehensive online intervention program.

ED is a refractory disorder with high mortality and recurrence, people who undertake the primary care work of these patients are faced with intense and severe challenges [3]. A number of studies before the COVID-19 pandemic have shown that the primary caregivers of the ED patients are subjected to great burden and psychological pressure, impaired family functions, poor quality of life, and serious emotional problems [6–9]. When managing ED patients’ daily diet and seeking medical assistance, the primary caregivers often have emotional symptoms such as anxiety and depression, and the intensity of undesirable emotions even exceeds the ED patients’ own self-reports [10, 11].

This study was desired to investigate the psychological distress and potential influencing factors of the primary caregivers of ED offspring during the COVID-19 pandemic. We hypothesized that during the COVID-19 pandemic, the primary caregivers of ED offspring experienced greater emotional pain, stress, and conflicts with family members than the primary caregivers of healthy offspring.

## Methods

### Study implementation

From March 6 to April 20, 2020, 378 questionnaires for primary caregivers of ED offspring and 1163 questionnaires for primary caregivers of healthy offspring were collected through an online crowdsourcing platform in mainland China. This study was approved by the Ethics Committee of the Shanghai Mental Health Center (SMHC) (2020–32), and all participants signed the informed consent to the study.

### Participants

Participants were recruited and data collected by SMHC Eating Disorder Treatment Center.

Inclusion criteria for primary caregivers of ED offspring: At least one offspring in the family who is definitely diagnosed with ED or suspected ED; The offspring has active ED symptoms during the COVID-19 pandemic; The caregiver is father or mother, the person in the family who primarily cares for and lived with the ED offspring during the COVID-19 pandemic. Exclusion criteria for primary caregivers of ED offspring: The ED offspring has other serious mental illness or chronic physical disorders. The caregiver is caring for other family member with severe mental illness or chronic physical disorders.

Inclusion criteria for primary caregivers of healthy offspring: No offspring with physical or mental illness; The caregiver is father or mother, the person in the family who primarily cares for and lived with the healthy offspring during the COVID-19 pandemic. Exclusion criteria for primary caregivers of healthy offspring: The caregiver is taking care of other family member with severe mental illness or chronic physical disorders.

**Fig. 1:**
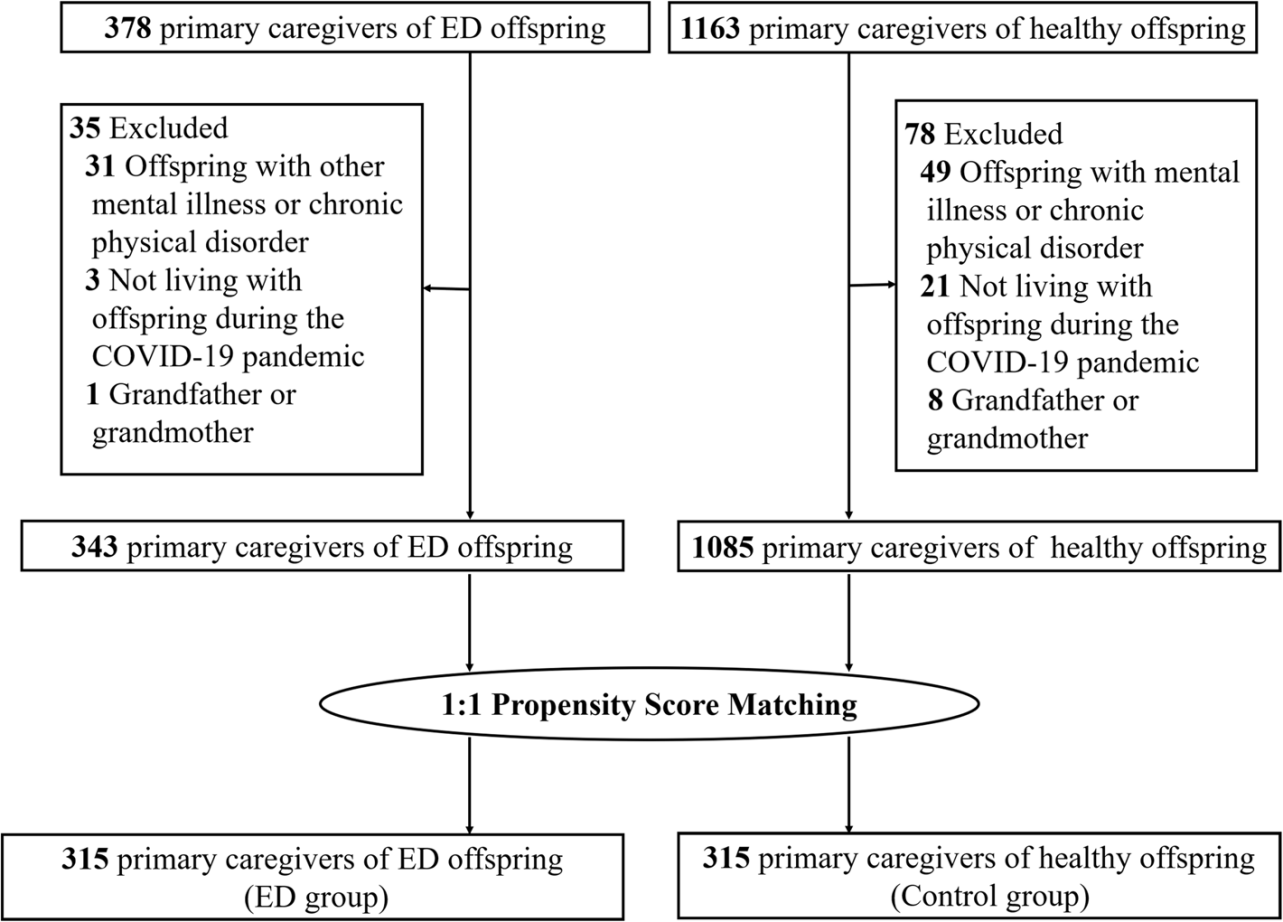
Study flowchart

Valid questionnaires that met the criteria included 343 (90.74%) primary caregivers of ED offspring and 1085 (93.29%) primary caregivers of healthy offspring. Using Propensity Score Matching (PSM), 315 (83.33%) primary caregivers of ED offspring (ED group) and 315 matched primary caregivers of healthy offspring (Control group) were included in the statistical analysis (shown in Fig. 1).

### Measurements

#### Demographic information

The ED group: Personal characteristics (age, gender, years of education, previous or present physical illness, previous or present mental illness, weight dissatisfied, body shape dissatisfied); Offspring information (age, gender, whether the ED offspring was definitely diagnosed, diagnostic subtypes); Family information [economic level, housing area per capita, marital satisfaction (0–100 score), intimacy with offspring (0–100 score), place of residence during the COVID-19 pandemic, family conflicts during the COVID-19 pandemic, family member diagnosed with COVID-19, isolation caused by the COVID-19, parenting style, the interaction time with offspring before and during the COVID-19 pandemic], etc.

The Control group: The information is the same as above, except that and materials related to the offspring’s ED diagnosis was removed.

#### Assessments scales

##### Patient health Questionnaire-9 (PHQ-9) [12]

PHQ-9 measures the depressive symptoms of individuals in the past 2 weeks, a total of 9 items. For each item, participants need to choose one of “nothing”, “a few days”, “more than half of the days” and “almost every day”, which are recorded as 0, 1, 2, and 3 points, respectively. This scale has good reliability and validity in the Chinese general hospital outpatients [13]. In this study, the Cronbach’s *α* was 0.892 in the ED group and was 0.830 in the Control group.

##### Generalized anxiety Disorder-7 (GAD-7) [14]

GAD-7 measures the individual’s anxiety status in the past 2 weeks, a total of 7 items. For each item, participants need to choose one of “never”, “occasional days”, “often, there is more than one week in the past two weeks” and “almost every day”, which are recorded as 0, 1, 2, 3 points, respectively. This scale has good reliability and validity in the Chinese cervical spondylosis patients [15]. In this study, the Cronbach’s *α* was 0.918 in the ED group and was 0.916 in the Control group.

##### Perceived stress Scale-10 (PSS-10) [16]

PSS-10 measures the situation in which the individual felt uncontrollable or overwhelmed in the past four weeks, a total of 10 items. Each item with a 5-point scale from 0 (never) to 4 (very common). The 4th item, the 5th item, the 7th item and the 8th item are reverse scoring. This scale has good reliability and validity in the Chinese psychological department patients [17]. In this study, the Cronbach’s *α* was 0.802 in the ED group and was 0.826 in the Control group.

##### Social support rating scale (SSRS)

SSRS was developed by Chinese scholar ShuiYuan Xiao [18]: Measuring the individual ‘s support in society, a total of 10 items. There are three subscales: objective support, subjective support, and utilization of support. This scale has good reliability and validity in the Chinese mental workers [19]. In this study, the Cronbach’s *α* of the total scale was 0.816 in the ED group and was 0.780 in the Control group.

### Statistical analysis

Use IBM® SPSS® software (Version 22.0) for data analysis. PSM was used for 1: 1 matching, group (ED group or Control group) as the dependent variable, primary caregivers’ age, and primary caregivers’ gender as independent variables for logistic regression, matching tolerance was 0.02, using nearest neighbor matching method and without replacement sampling method. One-Sample Kolmogorov-Smirnov test was used to perform normality test. Cross tab with Chi-square and Mann-Whitney U tests were used to compare the participants’ demographic variables and psychological distress of the two groups. The Kruskal-Wallis H test, Mann-Whitney U test, and Post Hoc tests with Bonferroni adjustment were used to perform univariate analysis on the total score of each scale corresponding to different demographic variables in the ED group. Ordinal logistic regression was used to perform regression analysis on the variables with statistical significance in the univariate analysis, housing area per capita, and years of education, and to examine the impact of each variable on the severity of depression and anxiety in the primary caregivers of ED offspring.

## Results

### Comparison of demographic variables between the ED group and the control group

According to the PSM results, there were 199 exact matches and 116 fuzzy matches. Thus, a total of 315 pairs were successfully matched. After matching, there were no significant differences between the ED group and the Control group in terms of age, gender, previous or present physical illness, and family member diagnosed with COVID-19. There were significant differences in other demographic variables.

As shown in Table 1, there were significantly more people in the ED group who had or were suffering from mental illness than the Control group. The education years of the ED group was significantly more than that of the Control group. Significantly fewer people with a low level and more people with medium or high economic levels in the ED group than that in the Control group. The housing area per capita of the ED group was significantly higher than that of the Control group. Marital satisfaction and intimacy with offspring in the ED group were significantly lower than that in the Control group. In the ED group, more parents adopted authoritarian style, and less parents adopted democratic style. Meanwhile, ED offspring’ parents have more family conflicts during the COVID-19 pandemic, and less interaction time with offspring before and during the COVID-19 pandemic.

**Table 1:** Comparison of demographic variables between the ED group and the Control group

Variables				***Md***(Q3-Q1)/***n***(%)		***Z***/***χ***^**2**^	***P***
				ED group (***n*** = 315)	Control group (***n*** = 315)		
**1. Personal characteristics**							
Age (years)				46 (49–44)	46 (49–44)	−0.074	0.941
Gender			Female	251 (79.7)	253 (80.3)	0.040	0.842
			Male	64 (20.3)	62 (19.7)		
Years of education				15 (16–12)	12 (14–9)	**−10.997**	**< 0.001**
Psychical illness			No	299 (94.9)	304 (96.5)	0.967	0.325
			Yes	16 (5.1)	11 (3.5)		
Mental illness			No	277 (87.9)	300 (95.2)	**10.898**	**0.001**
			Yes	38 (12.1)	15 (4.8)		
Weight dissatisfied			No	204 (64.8)	–	–	–
			Yes	111 (35.2)			
Body shape dissatisfied			No	221 (70.2)	–	–	–
			Yes	94 (29.8)			
**2. Offspring information**							
Age (years)				18 (21–16)	17 (19–13)	**−6.308**	**< 0.001**
Gender		Female		286 (90.8)	199 (63.2)	**67.806**	**< 0.001**
		Male		29 (9.2)	116 (36.8)		
Definitely diagnosed with ED		No		53 (16.8)	–	–	–
		Yes	AN	140 (44.4)			
			BN	77 (24.4)			
			BED	28 (8.9)			
			EDNOS	17 (5.4)			
Diagnostic subtypes	AN	Age (years)		17 (20–15)	–	–	–
		Gender	Female	129 (49.2)			
			Male	11 (4.2)			
	BN	Age (years)		20 (22.5–18)	–	–	–
		Gender	Female	75 (28.6)			
			Male	2 (0.8)			
	BED	Age (years)		19.5 (22–18)	–	–	–
		Gender	Female	28 (10.7)			
			Male	0 (0)			
	EDNOS	Age (years)		17 (21.5–16)	–	–	–
		Gender	Female	16 (6.1)			
			Male	1 (0.4)			
**3. Family information**							
Economic level			Low	66 (21.0)	113 (35.9)	**25.745**	**< 0.001**
			Medium	233 (74.0)	200 (63.5)		
			High	16 (5.1)	2 (0.6)		
Housing area per capita (m^2^)				37 (46.67–30)	30 (37.5–22)	**−7.018**	**< 0.001**
Marital satisfaction (0–100)				79 (85–60)	86 (100–79)	**−7.647**	**< 0.001**
Intimacy with offspring (0–100)				70 (82–53)	85 (100–80)	**−10.940**	**< 0.001**
Place of residence during the COVID-19 pandemic			Central China	17 (5.4)	40 (12.7)	**10.204**	**0.001**
			Non-central China	298 (94.6)	275 (87.3)		
Family conflicts			No	173 (54.9)	265 (84.1)	**63.408**	**< 0.001**
			Yes	142 (45.1)	50 (15.9)		
Diagnosed with COVID-19			No	314 (99.7)	314 (99.7)	0.000	1.000
			Yes	1 (0.3)	1 (0.3)		
Isolation caused by COVID-19			No	304 (96.5)	293 (93.0)	**3.869**	**0.049**
			Yes	11 (3.5)	22 (7.0)		
Parenting style			Authoritative	90 (28.6)	77 (24.4)	**23.275**	**< 0.001**
			Authoritarian	40 (12.7)	14 (4.4)		
			Democratic	159 (50.5)	209 (66.3)		
			Permissive	26 (8.3)	15 (4.8)		
Interaction time with offspring **before** the COVID-19 pandemic (minutes/day)				60 (120–30)	60 (150–30)	**−3.359**	**0.001**
Interaction time with offspring **during** the COVID-19 pandemic (minutes/day)				110 (200–31.25)	180 (300–60)	**−4.684**	**< 0.001**

*Note. ED* Eating Disorder, *Md* Median, *Q3* The Upper Quartile, *Q1* The Lower Quartile, *n* Number of samples, *Z Z*-Value, *χ*^2^ *χ*^2^-Value, *P P*-Value, *COVID-19* Coronavirus Disease 2019, *AN* Anorexia Nervosa, *BN* Bulimia Nervosa, *BED* Binge Eating Disorder, *EDNOS* Eating Disorder not Otherwise Specified

### Comparison of the depression, anxiety, perceived stress and social support between the ED group and the control group

As shown in Table 2, the depression, anxiety and perceived stress in the ED group were significantly higher than those in the Control group (all *P* < 0.001), and social support was significantly lower than the Control group (*P* < 0.001).

**Table 2:** Comparison of total scores of PHQ-9, GAD-7, PSS-10, SSRS in the ED group and the Control group(*n* = 315)

Variables	***Md***(Q3-Q1)		***Z***	***P***
	ED group	Control group		
PHQ-9 total scores	5 (8–3)	2 (6–0)	**−8.129**	**< 0.001**
GAD-7 total scores	4 (7–2)	1 (4–0)	**−9.705**	**< 0.001**
PSS-10 total scores	18 (21–13)	13 (16–9)	**−9.689**	**< 0.001**
SSRS total scores	38 (44–32)	45 (50–39)	**−9.809**	**< 0.001**

*Note. ED* Eating Disorder, *Md* Median, *Q3* The Upper Quartile, *Q1* The Lower Quartile, *Z Z*-Value, *P P*-Value, *PHQ-9* The Patient Health Questionnaire-9, *GAD-7* Generalized Anxiety Disorder-7, *PSS-10* Perceived Stress Scale-10, *SSRS* Social Support Rating Scale, *n* Number of samples

The severity of depression and anxiety between two groups were significant different (shown in Table 3). Taking total scores of PHQ-9 and GAD-7 as greater than or equal to 10 as the cutoff point of depression [13] and anxiety [15], the rate of depression in the ED group (20.6%) was significantly higher than that in the Control group (4.1%), *χ*^2^ = 39.565, *P* < 0.001; The rate of anxiety in the ED group (16.5%) was significantly higher than that in the Control group (2.2%), *χ*^2^ = 37.868, *P* < 0.001.

**Table 3:** Comparison of the distribution of depression and anxiety severity between the ED group and the Control group

Variables		***n***(%)		*χ*^2^	*P*
		ED group (***n*** = 315)	Control group (***n*** = 315)		
Severity of depression	None (0–4)	135 (42.9)	212 (67.3)	**56.218**	**< 0.001**
	Mild (5–9)	115 (36.5)	90 (28.6)		
	Moderate (10–14)	40 (12.7)	11 (3.5)		
	Severe (15–27)	25 (7.9)	2 (0.6)		
Severity of anxiety	None (0–4)	170 (54.0)	245 (77.8)	**53.663**	**< 0.001**
	Mild (5–9)	93 (29.5)	63 (20.0)		
	Moderate (10–14)	39 (12.4)	5 (1.6)		
	Severe (15–21)	13 (4.1)	2 (0.6)		

*Note. ED* Eating Disorder, *n* Number of samples, *χ*^2^ *χ*^2^-Value, *P P*-Value

### ED group data analysis results

#### Univariate analysis

Based on the mean scores, marital satisfaction and intimacy with offspring were divided into “low” and “high” groups, and the interaction time with offspring before and during the COVID-19 pandemic were divided into “less” and “more” groups.

PHQ-9, GAD-7, PSS-10, SSRS total scores were set as dependent variables. Gender, previous or present physical illness, previous or present mental illness, family conflicts, economic level, marital satisfaction, intimacy with offspring, interaction time with offspring before and during the COVID-19 pandemic were independent variables.

Univariate analysis showed that females’ perceived stress was significantly higher than that of males (*P* = 0.008). Depression, anxiety and perceived stress of those with physical illness or mental illness were significantly higher than those without physical illness/mental illness (all *P* < 0.05, *all P* < 0.001). Social support of ED parents with mental illness was significantly lower than those without mental illness (*P* = 0.001). ED parents with weight dissatisfaction or body shape dissatisfaction had significantly higher depression (*P* = 0.027, *P* = 0.026).

There were significant differences in depression, perceived stress and social support among people with different economic levels (*P* = 0.005, *P* = 0.038, *P* < 0.001). Depression and perceived stress were significantly higher among those with a low economic level than those with a moderate economic level (*P* = 0.004, *P* = 0.045), while their social support was significantly lower than those with a moderate economic level (*P* < 0.001).

Depression, anxiety and perceived stress of ED parents with low marriage satisfaction were significantly higher (*P* < 0.001, *P* = 0.001, *P* = 0.021), and their social support was significantly lower than those with high marriage satisfaction (*P* < 0.001). Depression of those had low intimacy with offspring was significantly higher (*P* = 0.021), and their social support was significantly lower than those had high intimacy with ED offspring (*P* = 0.001). The depression and anxiety of those with family conflicts were significantly higher than those without family conflicts (all *P* < 0.001). During the COVID-19 pandemic, the social support of those with less interaction time with ED offspring was significantly lower (*P* = 0.001).

The depression score among the ED offspring’s diagnostic subtypes was significant different (*P* = 0.005). Depression of AN caregivers was significantly higher than that of BED caregivers (*P* = 0.004) (shown in Table 4).

**Table 4:** Comparison of total scores of PHQ-9, GAD-7, PSS-10, SSRS on different demographic variables in the ED group (*n* = 315)

Variables		PHQ-9 total scores			GAD-7 total scores			PSS-10 total scores			SSRS total scores		
		***Md***(Q3-Q1)	***Z***/***H***	***P***	***Md***(Q3-Q1)	***Z***/***H***	***P***	***Md***(Q3-Q1)	***Z***/***H***	***P***	***Md***(Q3-Q1)	***Z***/***H***	***P***
**1. Personal characteristics**													
Gender	Female	6 (9–3)	−0.401	0.688	4 (8–2)	−0.882	0.378	18 (22–14)	**−2.654**	**0.008**	38 (44–32)	− 0.504	0.614
	Male	5 (8–3)			4 (6–2)			16 (19–12)			38 (43–32)		
Psychical illness	No	5 (8–2)	**−2.617**	**0.009**	4 (7–2)	**−2.381**	**0.017**	18 (21–13)	**−1.994**	**0.046**	38 (44–32)	− 1.641	0.101
	Yes	8.5 (12.25–4.25)			7.5 (14–3.25)			20 (24.5–15.25)			34 (40.25–31.25)		
Mental illness	No	5 (8–2)	**−5.174**	**< 0.001**	4 (7–2)	**−4.174**	**< 0.001**	17 (21–12.50)	**− 3.960**	**< 0.001**	38 (45–32)	**−3.333**	**0.001**
	Yes	10 (14.25–6)			7 (12.25–4)			20.5 (25–17.75)			34 (38.25–27.75)		
Weight dissatisfied	No	5 (8–2)	**−2.213**	**0.027**	4 (7–2)	−1.898	0.058	17 (20.75–13)	− 1.927	0.054	37.5 (44.75–31)	−0.130	0.896
	Yes	6 (9–3)			5 (8–2)			18 (23–14)			38 (43–33)		
Body shape dissatisfied	No	5 (8–3)	**−2.225**	**0.026**	4 (7–2)	−1.546	0.122	18 (21–13)	− 1.344	0.179	38 (43–32)	−0.614	0.539
	Yes	7 (10–2.75)			5 (9–2)			18 (22–14)			38 (45–31.75)		
**2. Offspring information**													
Definitely diagnosed with ED	No	6 (10–2)	−0.364	0.716	4 (8–2)	− 0.150	0.881	16 (21–11)	−1.544	0.123	38 (42.5–32.5)	−0.008	0.993
	Yes	5 (8–3)			4 (7–2)			18 (21–14)			38 (44–32)		
Diagnostic subtypes	AN	6 (8.75–3)	**12.779**	**0.005**	4 (8–2)	3.382	0.336	18 (22–14)	5.180	0.159	37 (43–32)	3.992	0.262
	BN	5 (8–2.5)			4 (7–2)			18 (21–14)			39 (45–31.5)		
	BED	3 (5–1.25)			3.5 (5–2)			16 (20–12)			40 (45–34.5)		
	EDNOS	4 (8–1.5)			4 (9–0.5)			15 (21.5–11)			35 (42–30.5)		
**3. Family information**													
Economic level	Low	7 (10–3.75)	**10.550**	**0.005**	6 (10–2)	4.298	0.117	20 (23–14.75)	**6.535**	**0.038**	33 (38.25–29)	**17.048**	**< 0.001**
	Medium	5 (8–2)			4 (7–2)			17 (20–13)			39 (45–33)		
	High	6.5 (11–2.25)			4.5 (8.5–1)			18 (23–14.25)			35 (45–30.25)		
Marital satisfaction	Low	6 (10–4)	**−3.740**	**< 0.001**	6 (9–3)	**−3.360**	**0.001**	18 (22–15)	**−2.314**	**0.021**	33 (40–29)	**−5.550**	**< 0.001**
	High	4 (7–2)			4 (6.75–2)			17 (21–12)			40 (46–35)		
Intimacy with offspring	Low	6 (9.25–3)	**−2.306**	**0.021**	4.5 (8–2)	−1.499	0.134	18 (22–13.75)	−0.760	0.447	36 (42–30)	**−3.338**	**0.001**
	High	5 (8–2)			4 (7–2)			18 (21–13)			39 (45–33)		
Parenting style	Authoritative	6 (10–3)	5.148	0.161	5 (8–3)	6.017	0.111	18.5 (23–13)	4.365	0.225	38 (45.25–33)	4.638	0.200
	Authoritarian	6 (8–2)			5 (8.5–2)			19.5 (22.75–15.25)			38 (43–30)		
	Democratic	5 (8–2)			4 (7–2)			17 (21–13)			38 (44–32)		
	Permissive	5.5 (10.25–3)			4 (7–1)			18 (20–15)			35 (40.25–30)		
Place of residence during the pandemic	Central China	6 (11–4)	−1.223	0.221	4 (8.5–3.5)	−0.954	0.340	18 (23–15.5)	− 0.751	0.452	40 (43–37.5)	− 1.204	0.228
	Non-central China	5 (8–2.75)			4 (7–2)			18 (21–13)			38 (44–32)		
Family conflicts	No	4 (7–2)	**−4.980**	**< 0.001**	4 (6–1)	**−4.432**	**< 0.001**	17 (20–13)	−1.917	0.055	38 (45–32.5)	− 1.837	0.066
	Yes	7 (10–4)			6 (9.25–3)			18 (23–13)			36.5 (43–31)		
Diagnosed with COVID-19	No	5 (8.25–3)	−0.767	0.443	4 (7–2)	−1.469	0.142	18 (21–13)	− 1.344	0.179	38 (44–32)	−0.842	0.400
	Yes	–			–			–			–		
Isolation caused by COVID-19	No	5 (8–2)	−1.707	0.088	4 (7–2)	− 1.623	0.105	18 (21–13)	−0.653	0.514	38 (43–32)	− 0.642	0.521
	Yes	8 (9–4)			7 (9–3)			19 (24–13)			41 (48–30)		
Interaction time with offspring **before** the pandemic	Less	5 (9–3)	−0.482	0.630	4 (8–2)	−1.353	0.176	18 (21–13)	−0.463	0.643	38 (43–31)	−0.859	0.390
	More	6 (8–2)			4 (6.5–1)			18 (21–12)			38 (45–32.5)		
Interaction time with offspring **during** the pandemic	Less	6 (9–3)	− 1.382	0.167	4 (7–2)	− 1.512	0.130	17 (21–13)	−0.934	0.350	36.5 (43–30.25)	**−3.235**	**0.001**
	More	5 (8–2)			4 (7–2)			18 (22–13)			40 (46–34)		

*Note. ED* Eating Disorder, *Md* Median, *Q3* The Upper Quartile, *Q1* The Lower Quartile, *Z Z*-Value, *H H*-Value, *P P*-Value, *PHQ-9* The Patient Health Questionnaire-9, *GAD-7* Generalized Anxiety Disorder-7, *PSS-10* Perceived Stress Scale-10, *SSRS* Social Support Rating Scale, *COVID-19* Coronavirus Disease 2019, *AN* Anorexia Nervosa, *BN* Bulimia Nervosa, *BED* Binge Eating Disorder, *EDNOS* Eating Disorder not Otherwise Specified, *n* Number of samples

#### Ordinal logistic regression analysis of the severity of depression and anxiety

According to PHQ-9 and GAD-7 total scores, the severity of depression and anxiety is divided into four levels: None (0–4), mild (5–9), moderate (10–14) and severe (15 points and above), with the severity of anxiety and depression as the dependent variables, with PSS-10 total scores, SSRS total scores, variables with statistical differences in univariate analysis (previous or present physical illness, previous or present mental illness, weight dissatisfied, body shape dissatisfied, patient’s diagnostic subtypes, family conflicts, economic level, marital satisfaction and intimacy with offspring), housing area per capita and years of education as the independent variables.

Ordinal logistic regression analysis showed that perceived stress, social support, previous or present mental illness, and family conflicts had significant effects on the severity of depression. Among them, perceived stress is a risk factor (*OR* = 1.314), high social support, no mental illness and no family conflicts are protective factors (*OR* = 0.942, *OR* = 0.430, *OR* = 0.495). (shown in Table 5).

**Table 5:** Ordinal logistic regression with depression severity as the dependent variable (*n* = 262)

Variables		***B***	***SE***	***Wald***	***P***	***OR***	95%***CI***	
							***Lower***	***Upper***
Physical illness	No	−0.022	0.608	0.001	0.971	0.978	−1.213	1.169
	Yes	0				1		
Mental illness	No	−0.845	0.413	4.191	**0.041**	**0.430**	−1.654	−0.036
	Yes	0				1		
Family conflicts	No	−0.704	0.286	6.042	**0.014**	**0.495**	−1.265	−0.143
	Yes	0				1		
Economic level	Low	0.176	0.674	0.068	0.794	1.192	−1.146	1.497
	Medium	−0.156	0.606	0.066	0.797	0.856	−1.344	1.032
	High	0				1		
Marital satisfaction	Low	0.045	0.288	0.024	0.877	1.046	−0.521	0.610
	High	0				1		
Intimacy with offspring	Low	−0.345	0.336	1.058	0.304	1.148	−1.003	0.313
	High	0				1		
Weight dissatisfied	No	−0.345	0.336	1.058	0.304	0.708	−1.003	0.313
	Yes	0				1		
Body shape dissatisfied	No	−0.233	0.359	0.423	0.515	0.792	0.937	0.470
	Yes	0				1		
Patient’s diagnostic subtypes	AN	0.754	0.616	1.498	0.221	2.125	−0.453	1.961
	BN	0.612	0.634	0.931	0.335	1.844	−0.631	1.854
	BED	0.005	0.737	0.000	0.994	1.005	−1.440	1.450
	EDNOS	0				1		
Housing area per capita		−0.008	0.008	0.979	0.323	0.992	−0.032	0.007
PSS-10 total scores		0.273	0.033	67.701	**< 0.001**	**1.314**	0.208	0.338
SSRS total scores		−0.059	0.019	9.767	**0.002**	**0.942**	−0.097	−0.022

*Note. B* Beta-Value, *SE* Standard Error, *Wald Wald*-Value, *P P*-Value, *OR* Odds Ratio, *CI* Confidence Interval, AN Anorexia Nervosa, *BN* Bulimia Nervosa, *BED* Binge Eating Disorder, *EDNOS* Eating Disorder not Otherwise Specified, *n* number of samples

Perceived stress, social support, family conflicts, and years of education had significant effects on the severity of anxiety. Among them, perceived stress is a risk factor (*OR* = 1.444), high social support, no family conflicts, and high years of education are protective factors (*OR* were 0.946, 0.417 and 0.899 respectively) (Shown in Table 6).

**Table 6:** Ordinal logistic regression with anxiety severity as the dependent variable (*n* = 315)

Variables		***B***	***SE***	***Wald***	***P***	***OR***	95%***CI***	
							***Lower***	***Upper***
Physical illness	No	−0.557	0.546	1.039	0.308	0.573	0.197	1.671
	Yes	0				1		
Mental illness	No	−0.270	0.376	0.517	0.472	0.763	0.365	1.595
	Yes	0				1		
Family conflicts	No	−0.874	0.279	9.802	**0.002**	**0.417**	0.241	0.721
	Yes	0				1		
Marital satisfaction	Low	0.043	0.282	0.024	0.878	1.044	0.601	1.814
	High	0				1		
Years of education		−0.106	0.036	8.968	**0.003**	**0.899**	0.838	0.964
PSS-10 total scores		0.368	0.037	98.079	**< 0.001**	**1.444**	1.343	1.553
SSRS total scores		−0.056	0.018	9.347	**0.002**	**0.946**	0.912	0.980
Housing area per capita		−0.003	0.007	0.198	0.656	0.997	0.983	1.011

*Note. B* Beta-Value, *SE* Standard Error, *Wald* Wald-Value, *P P*-Value, *OR* Odds Ratio, *CI* Confidence Interval, *n* number of samples

## Discussion

### The rates of depression and anxiety in the ED group and the control group

The primary caregivers of ED offspring during the COVID-19 pandemic experienced greater depression and anxiety than the primary caregivers of healthy offspring. The depression rate in ED group was 20.6%, which was comparable to the data of a previous Meta-analysis study (18.9%) [20] and was significantly higher than Control group (4.1%). The anxiety rate in ED group was 16.5%, which was comparable to the data of the previous two studies [anxiety rates were 16.51% [21] and 12.09% [22], respectively] and was significantly higher than those of primary caregivers of healthy offspring (2.2%). The psychological distress of the primary caregivers of ED offspring during the COVID-19 was sever.

### Relationship between personal characteristics and psychological distress of primary caregivers of ED offspring

In ED group, females (79.7%), as the primary caregivers of ED, have a higher perceived stress during the pandemic compared to males, similar to previous studies [23]. Females are more emotionally sensitive, may have higher perceived stress, and bear greater burden in dealing with their offspring. It is suggested that we should pay special attention to mother’s mental health, and it is necessary to increase father’s participation and emotional support for the mother during future intervention.

The higher years of education in the ED group, the lower the severity of anxiety. Based on our clinical experience we speculate that there are two possible explanations. Firstly, people with more education may have a stronger ability to screen information and assess the pandemic situation more objectively, thus experienced less anxiety themselves. Secondly, they may have invested more in family relationships, and effectively helped their offspring dealing with ED symptoms, thereby having less family stress. But more specific studies are needed to verify these speculations.

The proportion of ED parents with weight dissatisfaction and body shape dissatisfaction in the ED group was 35.2 and 25.8%, respectively. Those with weight dissatisfaction had higher depression than those without weight dissatisfaction, and those with body shape dissatisfaction had higher depression score, which similar to previous research results [24]. Many studies have suggested that there are certain associations between family eating patterns, mother’s ED symptoms, and offspring’ unhealthy eating problems [25, 26], the greater offspring’ perceived pressure from mother, the more symptoms of ED [27]. The primary caregiver’s weight loss behavior, weight dissatisfaction, and body shape dissatisfaction are likely to affect the offspring’ perception of their own body and thus develop into one of the susceptible factors for ED.

### Relationship between offspring’s diagnostic subtypes and the psychological distress of primary caregivers of ED offspring

All offspring in the ED group had active ED symptoms. The proportion of offspring definitely diagnosed with ED was 83.2% (AN: 53.4%; BN: 29.6%; BED: 10.7%; Other ED: 6.5%). The primary caregivers of AN was most depressive. A possible explanation is that compared with other types of ED, patients with AN are often accompanied by a variety of physical complications, severe malnutrition, and a very high risk of death, requiring multi-party cooperation for comprehensive intervention treatment [28]. However, during the COVID-19 pandemic, our ED treatment center was unable to provide inpatient and outpatient services due to the quarantine policy, the primary caregivers of AN cannot obtain the assistance of professional medical staff.

### Relationship between economic status, family interaction and the psychological distress of primary caregivers of ED offspring

The economic status of the ED group (economic level, housing area per capita) is significantly higher than that of the Control group. Our results are compatible with previous studies that have found that there is a certain correlation between social class and body shape concern, and symptoms of ED and body dissatisfaction in middle-class families and high-income families are more obvious [29, 30].

The number of people with family conflicts during the COVID-19 pandemic in the ED group was significantly higher than that in the Control group. Marriage satisfaction and intimacy with offspring were significantly lower than the Control group. The interaction time with offspring before and during the COVID-19 pandemic were significantly less than those in the Control group. During the COVID-19 pandemic, those with family conflicts and low marriage satisfaction have higher depression and anxiety, and those with low offspring intimacy have higher depression. Several previous studies have shown that family dysfunction can interfere with the mental health of offspring [31–33], meanwhile caring for offspring with ED can also be extremely stressful and painful for the entire family [34–37].

Quarantine increases the interaction time between parents and offspring. For families with relatively good family function, this may increase social support and intimacy with offspring, reducing depression and anxiety; For families with relatively family dysfunction, it may increase the possibility of conflicts, especially those with poor marriage quality and low family intimacy, they may have more psychological distress [38, 39].

### Potential bias of results

The methodology of crowd sourcing which can speed up data collection and reduce experimental cost, but it is faced with the risk of low data quality and poor representativeness. In our study, the age, income level and years of education of ED caregivers and healthy offspring caregivers were relatively low, and the number of people from Non-central China in the two groups and the number of AN caregivers in the ED group were significantly higher, which would affect the reliability and the degree of promotion of our research results. This study found that the psychological distress of ED caregivers is significantly higher than that of healthy offspring’s caregivers. It suggests that we should pay more attention to ED caregivers. However, due to the shortages of crowd sourcing, we have to treat the results with more caution. We hope that future researches can use more rigorous and detailed methods to expand the range of age, income, years of education, increase the number of BN caregivers, BED caregivers, and include more caregivers in central China.

### Limitations

Firstly, this study is a cross-sectional study, we didn’t collect data illustrating psychological distress in these ED caregivers before the COVID-19 pandemic. We have no way of knowing exactly what factors causes ED primary caregivers’ psychological distress during the COVID-19 pandemic, and we can’t conclude that these psychological distresses are unique to the COVID-19 pandemic.

Secondly, we fail to collect enough information about ED offspring, particularly about the illness severity among individuals with EDs. Although we found some differences in psychological distress among primary caregivers of eating disorders with different subtypes, we could not rule out the influence of illness severity.

Thirdly, all the data came from caregivers’ self-reports. The majority of caregivers’ offspring were definitely diagnosed with ED at SMHC Eating Disorder Treatment Center (according to DSM-5), but some offspring were not definitely diagnosed in psychiatric hospitals. There may be some ED offspring who have active symptoms but not full diagnosis during the COVID-19 pandemic. Although by comparing the definitely diagnosed group with the not definitely diagnosed group, we found that there was no significant difference in anxiety, depression, perceived stress and social support between the two groups, we can’t ignore the potential confusion.

Fourthly, the primary caregiver of ED is defined as father or mother in this study, while other caregivers are not included, our findings cannot be generalized to all primary caregivers of ED patients.

Fifthly, the ED group and the Control group cannot be completely matched in multiple demographic variables, and may also have a partial impact on the results.

### Outlook

There is a saying in China: Family is a sweet burden. This is especially true for ED families during the COVID-19 pandemic. The dual pressures of domestic strife and external stress may make the primary caregivers of ED offspring more emotional vulnerability and have more perceived stress. They need more professional assistance and psychological intervention. Considering that ground-based interventions are not available during the pandemic, it is recommended to provide various forms of network support and interventions for parents with ED offspring to improve their mental health, and help their ED offspring.

## Conclusions

During the COVID-19 pandemic, primary caregivers of ED offspring experienced more psychological distress than that of primary caregivers of healthy offspring. ED caregivers with high perceived stress may have higher levels of depression and anxiety. ED caregivers with high social support, no mental illness and no family conflicts may have lower levels of depression. ED caregivers with high social support, no family conflicts, and high years of education may have lower levels of anxiety.

## Data Availability

The datasets used and/or analyzed during the current study are available from the corresponding author on reasonable request.

## Abbreviations

COVID-19: Coronavirus Disease 2019
ED: Eating Disorder
AN: Anorexia Nervosa
BN: Bulimia Nervosa
BED: Binge Eating Disorder
EDNOS: Eating Disorder not Otherwise Specified
PSM: Propensity Score Matching
*Md*: Median
Q3: The Upper Quartile
Q1: The Lower Quartile
*n*: Number of samples
*Z*: *Z*-Value
*χ*^2^: *χ*^2^-Value
*P*: *P*-Value
*H*: *H*-Value
*B*: Beta-Value
*SE*: Standard Error
*Wald*: *Wald*-Value
*OR*: Odds Ratio
*CI*: Confidence Interval
PHQ-9: The Patient Health Questionnaire-9
GAD-7: Generalized Anxiety Disorder-7
PSS-10: Perceived Stress Scale-10
SSRS: Social Support Rating Scale
